# The impact of disasters on economic growth in selected Southern Africa development community countries

**DOI:** 10.4102/jamba.v13i1.1081

**Published:** 2021-10-29

**Authors:** Emmanuel Owusu-Sekyere, Wilfred Lunga, Selma T. Karuaihe

**Affiliations:** 1 Human Sciences Research Council, Pretoria, South Africa; 2 African Centre for Disaster Studies (ACDS), Potchefstroom, South Africa; 3 Department of Agriculture Economics, Extension and Rural Development, University of Pretoria, Pretoria, South Africa

**Keywords:** natural hazard, disasters, dynamic panel data econometrics, economic growth, South Africa, Southern African Development Community, SADC

## Abstract

This research study explores the impact of disasters on economic growth in selected Southern Africa Development Community countries. Annual data from 2005 to 2019 and panel data econometric estimation techniques are used in this study. The estimation approaches used control for both pooled and individual effects, heteroscedasticity, serial correlation, moderate levels of endogeneity and cross-sectional dependence (CSD). We found that although the impact of disasters on economic growth may be negative contemporaneously, reconstruction and recovery activities if well-resourced could facilitate building back better, which could ultimately lead to positive outcomes on economic growth a year after the disaster. We further tested the hypothesis in existing literature and confirm that quality institutions, favourable financial conditions and adequate access to international markets enhance a country’s coping and adaptive capabilities to disasters, thereby reducing the country’s level of risk to disasters.

## Introduction

Research studies on the impact of disasters on prone regions have gained considerable attention over time. This is driven by increased awareness of the disastrous impact of climate change and variability and the need for building back better post-disaster. For instance, in the Southern Africa Development Community (SADC), decades of interconnected disasters have claimed approximately 1.3 million lives between 1998 and 2017 (The World Bank 2019). There are no signs of the risk posed by natural hazards decreasing, particularly having witnessed the devastating effect of droughts, climate change-induced floods and cyclones in recent times (The World Bank 2019). Disaster scholarship has established that natural hazards can create or exacerbate disaster vulnerabilities (United Nations General Assembly 2017). Furthermore, when the disaster occurs in the region, its associated adverse effects stall development efforts at least in the immediate term. The lack of proper coordination leads to inefficient allocation of resources by separate government institutions for disaster risk reduction and climate change within SADC member states, duplication of services, polarisation of interventions, incoherent policies, competing for the same resources and territorial contests (Nemakonde et al. 2021). In addition, heterogeneity across SADC countries and populations may require differentiated policy interventions, which further complicate the appropriateness of policy responses to disasters, especially those with cross-border socio-economic impact and spillover effects. The United Nations International Strategy for Disaster Risk Reduction (UNISDR 2017) posited that understanding the economic impacts of disasters plays an important role in the effective mitigation of disaster damage, which is one of the key challenges facing nations located in disaster-prone regions that are aiming to achieve sustainable growth.

Today, climate change-induced natural hazards is one of Southern Africa’s main threats (Van Niekerk et al. 2019).

This research study, therefore, seeks to explore the impact of disasters on economic growth in selected SADC countries, a region highly prone to disasters of diverse forms. The aim of this study was to observe what the immediate effect on economic growth is and ideally which are the factors that can facilitate building back better. Disasters are measured using the World Risk Index (WRI), whilst economic growth is measured using annual percentage GDP growth. The regionalisation of the study is important because of the spillover effects of disasters in the form of either physical damage, economic impact or humanitarian crisis.

## Literature review

There are several perspectives reflecting the impact of disasters on the economy. According to the United Nations General Assembly (2017), disaster risk creation is a serious disruption of the functioning of a community or a society at any scale because of hazardous events that interacts with conditions of exposure, vulnerability and capacity. This leads to one or more of the following: human, material, economic and environmental losses and impacts (United Nations General Assembly 2017). Thus, the Sendai Framework for Disaster Risk Reduction (SFDRR) upholds the development and implementation of measures to reduce hazard exposure and vulnerability to disasters (SFDRR 2015). Albalate and Padró-Rosario (2018) stated that one of the challenges for the disaster risk management sector is to integrate losses that are difficult to monetise into loss estimation procedures to obtain a sound quantification of disaster impacts. Researchers have the impression that disasters are an inescapable economic event that occurs naturally and alters the economy because of their power.

Researchers have examined disaster events of various types, such as Hurricane Katrina in 2005 in the United States, the 2010 earthquake in Haiti, and closer home, cyclone Idai that affected the SADC region, looking at their impacts shortly after their occurrence (Nemakonde et al. 2021). Albala-Bertrand (1993) and other studies have examined the impacts of disasters on economic growth considering various combinations of factors, such as time frames, definition of severity, disaster types and country samples (e.g. Cavallo et al. 2013; Felbermayr & Gröschl 2014; Fomby, Ikeda & Loayza 2013; Heger, Julca & Paddison 2008; Klomp 2016). As explained by Rodriguez-Oreggia et al. (2013), and Strobl (2011), the short-run impact of disasters on economic growth is negative, on average, and is most compelling for developing countries and small economies. However, the long-run effect of disasters has not been explained, and yet, it is important to discuss the distinction between both timeframes, short run and long run, as the effects could vary depending on the strength of the country.

Beside the issue of timeframe, Kliesen (1994) categorises the impact of disasters into direct and indirect losses. Direct losses relate to losses to critical infrastructure (CI) that may include buildings, highways and infrastructure, crops/production (stock variables, quantities measured at a point in time), whilst indirect losses refer to secondary effects such as disruptions to service delivery, for example, transport, utilities, tourism, employment losses, lost output and revenue (flow variables, measured per unit of time) (Kliesen 1994). Although there are suggestions that indirect losses are ‘more of a possibility than a reality’ estimation of indirect effects of disasters have helped to assess community vulnerability, measures required for mitigation, suitable levels of disaster assistance and insurance liability (Albala-Bertrand 1993; Rose 2004). Evidence also shows that the impact on CI is determined by the nature of the disaster. Meteorological and climatic change induced disasters have been found to positively relate to higher long-run economic growth, whilst geological disasters have a negative impact on economic growth (Min, Kim & Lee 2020; Seoko 2019; Skidmore & Toya 2002). Specific reasons for this direction of causation between different types of disasters and economic growth are not clear in the literature.

Economic analysis of disasters has further shown that the net effect of a disaster of any kind on the economy is determined by the extent to which the positive impact of recovery and reconstruction activities cancels out the negative impact of initial damages (Albala-Bertrand 1993). Disasters impact negatively on production input and infrastructure (Felbermayr & Groschl 2013; Kliesen 1994; Noy 2009). This is also supported by recent events of climate-related disasters that are intensifying in Southern Africa, with significant impacts on mining populaces, CI, environments and ecosystems. Disasters of all kinds reduce the real GDP per capita on impact and harm development (Díaz et al. 2020; Felbermayr & Groschl 2013). Their findings are in line with the neoclassical growth theory, which argues that if a disaster destroys part of a country’s capital stock (direct effect), then the production possibility frontier shifts inwards leading to lower total output per capita (indirect or secondary effect).

In contrast to these adverse consequences of disasters, reconstruction spending can provide a boost to the domestic economy by replacing outdated technology and structures or infrastructure (Awotona & Donlan 2008; Skidmore & Toya 2002). Although disasters may reduce physical capital investment, they also create an opportunity to replace or update damaged capital stock, thereby encouraging the adoption of new technologies. This perspective reflecting the impact of disasters on the economy aligns with endogenous growth models, suggesting that disasters can promote higher rates of growth through renewed physical and human capital (Bello 2006; eds. Bennett et al. 2014; Fan 2013; Kliesen 1994; Skidmore & Toya 2002) The findings of these researchers emphasised that rebuilding activity usually generates both increased sales tax receipts and additional employment. However, this varies between rich and poor countries because of different levels of technological advancement. Furthermore, Fomby et al. (2013) conducted a study on the response of economic growth to disasters in several developed and developing countries. The aim was to estimate a mean response of GDP growth to disasters, such as earthquakes, floods, droughts and storms. Their results revealed that earthquakes did not have a significant effect on aggregate GDP growth or on agricultural growth, instead they found a positive mean response in non-agricultural growth in year 0 and year 1 of the event, leading to growth in value addition. They emphasised that the increase is because of reconstruction activities, such as public infrastructure, housing and farming that take place after an earthquake.

Felbermayr and Groschl (2013) argued that a lack of appropriate institutions, inadequate financial conditions or limited access to international markets may hamper the catching up process after a disaster. This is true for most poor countries, as evidenced from the impact of earthquakes and cyclone-induced floods in countries, such as Malawi, Mozambique and Zimbabwe (Meteorological Service Department 2019). Schumacher and Strobl (2011) and Bennett et al. (eds. 2014) showed that the larger the economic and human losses associated with disasters, the poorer a country is relative to rich or high-income countries. In addition, middle- and low-income countries have difficulty financing reconstruction after disasters because of a lack of appropriate counter-cyclical fiscal policies to implement effective government responses, coupled with inadequate insurance and re-insurance markets (Fan 2013; Noy 2009).

In addition, measures of economic and financial openness help in mitigating the negative effects of disasters on GDP per capita. Whilst financial openness helps to deal with recovery costs of catastrophes, the mitigating power of inclusive independent and efficient institutions are relevant complements to financial openness in addressing the impact of disasters (Felbermayr & Groschl 2013). These different perspectives indicate that the impact of disasters on an economy could be direct or indirect, dependent on the nature of the disaster (whether climatic or geological) and the net effect of reconstruction and recovery compared with the initial damage of the disaster. Reconstruction and recovery are further dependent on whether the country is rich or poor, its initial level of technology, its level of financial openness and institutional quality, which determines a country’s ability to raise capital to cover reconstruction costs through appropriate policy responses.

The objective of this research article, therefore, was to ascertain the impact of disasters on economic growth in selected SADC countries. The aim was to observe which of these mitigating factors discussed above drive this impact and whether the net effect is positive or negative. The rest of this article is structured as follows: third section discusses data and methodology, fourth section empirical results and fifth section concludes.

## Data and methodology

### Data

Annual data from 2005 to 2019 from the United Nations University-Environment and Human Security (UNU-EHS) and World Development indicators of the World Bank are used in this study. We consider five countries, such as Botswana, Namibia, Swaziland, Tanzania and Zambia, in the SADC region in addition to South Africa, whose economies are largely disaster prone.

### Disasters

The WRI is used as the measure of disasters in this study, which consists of four components: exposure to a disaster, susceptibility, coping capacity and adaptive capacity of countries. Exposure refers to entities likely to be affected by a disaster, which include individuals, resources, infrastructure, production, goods and services, ecosystems, and socioecological systems (UNU-EHS 2011). Susceptibility relates to the likelihood of suffering damages in the event of a disaster. The susceptibility criterion reflects the conditions of people living in a particular country and their level of vulnerability in the event of a disaster in terms of the quality of public infrastructure, housing conditions, nutrition, poverty and dependencies, economic capacity, and income distribution (UNU-EHS 2011). Coping capacity refers to the extent to which a country’s government and authorities are prepared to minimise and mitigate the negative impact of a disaster in terms of early warning systems, medical services, social networks, and material coverage. Adaptive capacity relates to what measures are being implemented to ensure that society is more resilient to and less vulnerable to disasters. Adaptation measures include education and research, environmental and ecosystem protection, adaptation strategies and investments (UNU-EHS 2011). Consequently, as per these four categories of the WRI, a high index means a country is more exposed to disasters, more susceptible to its occurrence, and has poor coping and adaptation capabilities. In contrast a low WRI implies that although a country might be exposed to the occurrence of disasters it is less susceptible to its damaging effect because it has developed efficient coping and adaptive capabilities in managing the incidence and negative impact of disasters. By its composition, the WRI fully encapsulates the different perspectives reflecting the impact of disasters on the economy, namely, direct and indirect effects, the extent to which recovery and reconstruction mitigate the initial negative impact of disasters and the ability of countries to recover and reconstruct, which determines the final or net effect of disasters. The WRI further covers mediating factors, such as institutional quality, which has been discussed in the literature as one of the factors responsible for determining the extent to which a country can raise its capital to finance reconstruction costs (Felbermayr & Groschl 2013).

### Economic growth

Consistent with growth theory, GDP growth rate is used as the measure of economic growth. The direct impact of disasters is on physical capital stock (*gfcf*) measured as gross fixed capital formation as a ratio to GDP. This causes the production possibility frontier of the country concerned to shift inwards leading to a temporary decline in output. The direct impact of disasters on the production technology, capital stock and infrastructure of the country concerned leads to a decline in output (Felbermayr & Groschl 2013). The size of the labour force is represented by population growth (*pop*) measured as the annual percentage growth rate of the population in each country (Skidmore & Toya 2002). Human capital development (*hcd*) is measured in this study using public expenditure on education as a percentage of government expenditure. The human capital development variable plays two roles in this study. The (*hcd*) variable aligns with endogenous growth models that broadly define capital to include human capital as the source of technological progress by which economies can sustain growth in the steady state (Mankiw, Romer & Weil 1992; Solow 1956). The human capital development variable further represents a component of a country’s adaptation strategy – research and development - which reduces a country’s level of risk to disasters. The degree of urbanisation in each country (*urb*) is measured by the percentage growth rate of the urban population. Urbanisation serves as a measure of the extent of industrialisation of a country, with the assumption that labour migrates from the rural areas to urban areas in search of employment and a better quality of life (Lipset 1959). This also captures the migrant nature of the labour force in the countries in this panel. These variables are relevant to cross country growth regressions (Levine & Renet 1992). Table 1 details the definitions and sources of the variables used in this study.

**TABLE 1:** Sources and definition of variables.

Symbol	Variable	Source	Definition
GDP	Economic growth	World Bank	Annual % GDP growth
GFCF	Capital stock	World Bank	Gross fixed capital formation as a ratio to GDP.
WRI	Natural disaster measure	UNU-EHS	World risk index
POP	Size of the economy	SARB	Percentage annual population growth
HCD	Human capital development	World Bank	Public expenditure on education as a percentage of government expenditure
POLITY	Institutional quality – Index of democracy	Polity IV	A combined score obtained by subtracting the autocracy score from the democracy score. It ranges from +10 (strongly democratic) to -10 (strongly autocratic). It is then normalised to one
FDI	Foreign direct investment	World Bank	Foreign direct investment as % of GDP
INT	Attractiveness to capital flows	World Bank	Real interest rate
URB	Degree of urbanisation	World Bank	Urban population rate (%) growth
KOPEN	Financial openness	World Bank	Chinn–Ito index of financial openness. Normalised to one.
Natural hazards × reconstruction	Interaction variable	Authors’ construction	Interaction variable of lagged natural disasters and reconstruction composite variable

WRI, World Risk Index; GDP, Gross Domestic Product; GFCF, Physical Capital Stock; POP, Population Growth; HCD, Human Capital Development; FDI, Foreign Direct Investment; KOPEN, Financial Openness; POLITY, Institutional quality; URB, Urbanisation in each country; INT, Interest rates; SARB, South Africa Reserve Bank; UNU-EHS, United Nations University-Environment and Human Security.

### Mediating factors

In addition to the growth model, a number of mitigating factors are considered in this study. This model includes a number of mitigating factors that are relevant in estimating the extent to which a country could raise international capital to cover reconstruction and recovery costs. These include an index of democratisation (*polity*) as a measure of institutional quality and foreign direct investment (*fdi*). The democracy index is a revised combined score that is computed by subtracting the autocracy score from the democracy score. The resulting unified polity score ranges from +10 (strongly democratic) to -10 (strongly autocratic) (Loayza et al. 2012; Noy 2009; Skidmore & Toya 2002).

Financial conditions are measured by the real interest rates (*rint*) and the degree of financial openness (*kopen*). The level of financial openness of each country is measured by the Chinn and Ito index (2007). The Chinn–Ito Index of financial openness measures how open a country is to cross-border capital transactions, ranging from an index of 2.44 (most financially open) to -1.86 (least financially open). The financial openness index and the real interest rate variables capture the ability of countries to attract capital flows for reconstruction and recovery post-disaster (Chinn & Ito 2007), which ultimately determines the net impact of natural disasters on an economy. These mediating factors further align with the argument of Felbermayr and Groschl (2013) that the lack of appropriate institutions, inadequate financial conditions or limited access to international markets may hamper the recovery process after a disaster.

### Cross-correlation analysis

Cross-correlation analysis of the variables is carried out in two batches. Table 2 depicts the cross-correlation between economic growth, disasters, and other determinants of growth as per endogenous growth models explained above. The results reveal a positive relationship between economic growth and disasters; however, the dynamics of time is not clear in correlation analysis whether this relationship is contemporaneous or asynchronous. Natural disasters are strongly positively correlated with population growth, indicating that the higher the population the higher the level of vulnerability to disasters. Human capital development and urbanisation are both negatively correlated with disasters. This indicates that the higher the level of research and development and industrialisation, the better a country’s adaptation and coping strategies towards the risk of disasters.

**TABLE 2:** Cross-correlation matrix of variables – Growth model.

Variables	GDP	WRI	GFCF	POP	HCD	URB
GDP	1.00	-	-	-	-	-
WRI	0.36***	1.00	-	-	-	-
GFCF	−0.04	0.09	1.00	-	-	-
POP	0.08	0.63***	0.51***	1.00	-	-
HCD	−0.20	−0.24**	0.13	−0.10	1.00	-
URB	0.05	−0.47***	0.12	−0.33***	−0.32***	1.00

WRI, World Risk Index; GDP, Gross Domestic Product; GFCF, Physical Capital Stock; POP, Population Growth; HCD, Human Capital Development; URB, Urbanisation in each country.

* , 10% level of significance;

** , 5% level of significance;

*** , 1% level of significance.

Table 3 shows the results of correlation analysis between disasters and the mediating factors found by Felbermayr and Groschl (2013) to help countries build back better from the incidence of disasters.

**TABLE 3:** Cross-correlation matrix of variables – mediating factors model.

Variables	WRI	POLITY	KOPEN	RINT	FDI	HCD
WRI	1.00	-	-	-	-	-
POLITY	−0.36***	1.00	-	-	-	-
KOPEN	−0.21**	0.45***	1.00	-	-	-
RINT	0.24**	0.09	0.09	1.00	-	-
FDI	0.21	0.22	0.11	−0.06	1.00	-
HCD	−0.24**	0.10	−0.02	−0.21	−0.08	1.00

WRI, World Risk Index; FDI, Foreign Direct Investment; KOPEN, Financial Openness; POLITY, Institutional quality; RINT, real interest rates; HCD, Human Capital Development.

* , 10% level of significance;

** , 5% level of significance;

*** , 1% level of significance.

**FIGURE 1:**
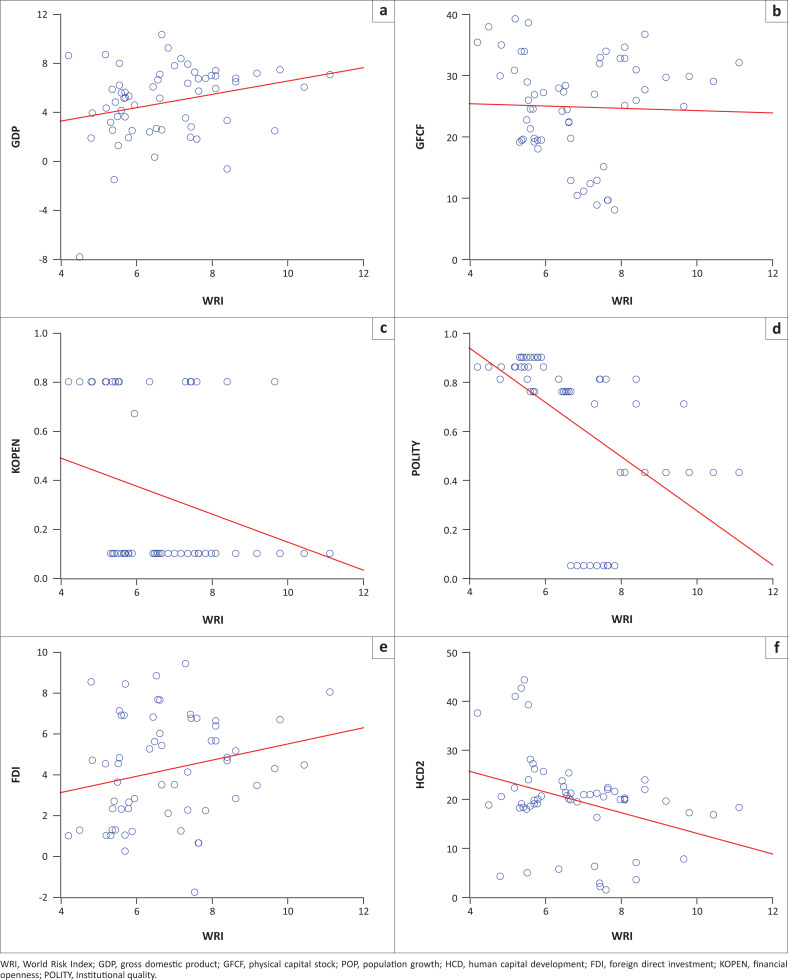
Scatter graphs of disasters and selected variables.

Institutional quality (*polity*), human capital development (*hcd*) and financial openness (*kopen*) are negatively related to natural disasters (*wri*). Attractiveness to capital flows as measured by the real interest rate and foreign direct investment are positively related to disasters. This is because the more attractive a country is to capital flows, the more foreign direct investment it can receive, and hence, the better positioned it is to reconstruct and recover after the incidence of a disaster. These initial indications from the cross-correlation analysis align with Felbermayr and Groschl (2013) that appropriate institutions and adequate financial conditions improve a country’s coping and adaptive capabilities, thereby reducing their level of risk to disasters. Scatter diagrams shown in Figure 1 depict a similar low positive relationship between disasters and economic growth, as shown by the cross-correlation analysis. Correlation does not necessarily mean causation, and therefore, an empirical estimation of the data would be useful for establishing the impact of disasters on economic growth in the countries studied.

### Model specification and estimation technique

Three estimations are of interest in this study. In the first estimation, a standard growth model is estimated to explore the contemporaneous relationship between disasters and economic growth. Secondly, we explore the asynchronous impact of disasters on economic growth by regressing economic growth on the lag of disasters. As the annual data are used in this study, the aim was to establish the impact of a disaster that was reported a year ago on economic growth today. Additionally, a composite variable called *reconstruction* is constructed using mediating factors found in research to facilitate recovery from the impact of disasters, which include financial openness, real interest rate, foreign direct investment, institutional quality and human capital development (Felbermayr & Groschl 2013). The *reconstruction* variable is created by principal component analysis in statistics data (STATA) software and is interacted with lag of disasters *lwri × reconstruction*, which is also regressed on economic growth a year after the disaster. This is to ascertain the relationship between a disaster a year ago interacted with recovery efforts and economic growth a year later. Thirdly, we estimate a model with disasters as the dependent variable and the mediating factors that mitigate the initial negative impact of disasters as independent variables. This is to test the hypothesis of Felbermayr and Groschl (2013) that appropriate institutions, adequate financial conditions and access to international markets enhance coping and adaptive capabilities of countries, thereby reducing their level of risk to disasters.

### The growth and natural hazards induced disaster model

Table 4 details the results of tests on the panel data characteristics of the dataset for the growth model. The results reveal that there are country-specific and time-specific effects that need to be taken into consideration in the estimation process. Country-specific characteristics would include differences in economic growth and the size of their economies, population sizes, rate of urbanisation, quality of human capital development and gross fixed capital formation. Time-specific characteristics refer to the fact that countries in this study have experienced different types of disasters at different time periods.

**TABLE 4:** Panel data characteristics of the dataset – the growth model.

Test	Test static	Critical/prob. value	Inference
Joint validity of cross-sectional individual effects: *H*_*0*_ : *μ*_*1*_ = *μ*_*2*_ … *μ*_*N-1*_ = *0* *H*_*A*_: *Not all equal to 0*	*F* stat = 2.51	*F* (0.05, 5, 73) = 2.34	*F* stat > *F* critical: There are country-specific effects.
Joint validity of time (period) fixed effects: *H*_*0*_: *λ*_*1*_ = … *λ*_*T-1*_ = *0* *H*_*A*_: *Not all equal to 0*	*F* stat = 2.30	*F* (0.05, 13, 65) = 1.87	*F* stat < *F* critical: There are time-specific effects.
Haussmann test: Nickel (1981) bias: *H*_*0*_ *:E(Xit,/uit) = 0* *H*_*0*_ *:E(Xit,/uit) ≠ 0*	$\chi_{5}^{2}$ = 13.10	Prob. = 0.04	We reject the H*o* that there is no endogeneity between the lag of the dependent variable and the fixed effect error term.
Haussmann specification test: other: *H*_*0*_: *E(Xit,/uit) = 0* *H*_*0*_: *E(Xit,/uit) ≠ 0*	$\chi_{5}^{2}$ = 33.91	Prob. = 0.00	We reject the Ho that there is no endogeneity between the regressors and the error term.
Breusch and Pagan (1980) LM test for cross-sectional dependence: *H*_*0*_: *corr (μi,t,μj,t) = 0 for i ≠ j* *H*_*A*_: *corr (μi,t,μj,t) ≠ 0* *for some i ≠ j*	CD = 30.94	Prob. = 0.01	Cross-sections are interdependent.

CD, critical difference; LM, Lagrange multiplier; Prob., probability.

The estimation involving the growth model is, therefore, specified in Equation 1 as follows:

$$rgdpc_{it}=\beta_{1}wri_{it}+\beta_{2}X_{it}+\mu_{i}+\lambda_{t}+\nu_{it},$$ [Eqn 1]

for *i = 1,… N; t = 1, … T,* where *i* denotes country and *t* denotes time, *µ* denotes country-specific effects, λ time-specific effects and ν the idiosyncratic error term. *X* is a vector of control variables given by *X* = [*wri, gfcf, pop, hcd, urb*]. All the variables in the model are defined in Table 1.

In the Hausmann tests for endogeneity, we fail to reject the null hypothesis of no endogeneity with and without the lag of the dependent variable, indicating multiple sources of endogeneity beyond a Nickel (1981) bias. The Breusch and Pagan (1980) Lagrange Multiplier (LM) test for cross-sectional dependence (CSD), applicable when *T > N,* indicates that the countries in the panel are interdependent. This can be explained by the fact that they are all SADC countries, have several regional and bilateral protocols between them, cross-border trade, similar cultures and identical economies.

Additionally, an estimation of data on countries located geographically in the same region warrants the need to control for spillover effects. Spillover effects of disasters on neighbouring countries could be in the form of physical damage or a humanitarian crisis. Consequently, in estimating the model on the relationship between economic growth and disasters, the:

[*E*]stimation approach used must control for country specific effects, time specific effects, endogeneity, cross sectional dependence of the error term in addition to the given assumptions of the classical linear regression model, i.e. serial correlation and heteroscedasticity. (Osarumwense 2021:96)

### Natural hazards induced disasters and mediating factors model

The results of tests reveal the panel data characteristics of the dataset for the mediating factors model, as shown in Table 5. For this model, there are neither a country-specific or time-specific effects,nor is there a Nickel (1981) bias source of endogeneity. However, there is endogeneity emanating from the regressors.

**TABLE 5:** Panel data characteristics of the dataset – mediating factors model.

Test	Test static	Critical/prob. value	Inference
Joint validity of cross-sectional individual effects: *H*_*0*_ : *μ*_*1*_ =*μ*_*2*_ … *μ_N-1_ = 0* *H*_*A*_ : *Not all equal to 0*	*F* stat = 1.45	*F* (0.05, 5, 72) = 2.34	*F* stat < *F* critical: There are no country-specific effects.
Joint validity of time (period) fixed effects: *H*_*0*_ : *λ*_*1*_= … *λ*_*T-1*_= *0* *H*_*A*_: *Not all equal to 0*	*F* stat = 0.41	*F* (0.05, 13, 65) = 1.87	*F* stat < *F* critical: There are no time-specific effects.
Haussmann test: Nickel (1981) bias: *H*_*0*_ *:E(Xit,/uit) = 0* *H*_*0*_ *:E(Xit,/uit) ≠ 0*	$\chi_{5}^{2}$ = 6.61	Prob. = 0.25	We fail to reject the H*o* that there is no endogeneity between the lag of the dependent variable and the fixed effect error term. No Nickel (1981) bias.
Haussmann specification test: other: *H*_*0*_ *:E(Xit,/uit) = 0* *H*_*0*_ *:E(Xit,/uit) ≠ 0*	$\chi_{5}^{2}$ = 25.19	Prob. = 0.00	We reject the Ho that there is no endogeneity between the regressors and the error term. Alternative sources of endogeneity and not Nickel (1981) bias.
Breusch and Pagan (1980) LM test for cross-sectional dependence: H_0_: corr (*μi,t,μj,t*) = 0 for i ≠*j* H_A_: corr (*μi,t,μj,t*) ≠ 0 *for some i ≠j*	CD = 38.51	Prob. = 0.010	Cross-sections are interdependent.

CD, critical difference; LM, Lagrange multiplier; Prob., probability.

The Breusch and Pagan (1980) LM test results reveal that the countries are interdependent. Thus, the key characteristics to provide for in the mediating factors model are endogeneity and CSD of the error term. Based on the results of initial diagnostics of the variables in the mediating factors model, the applicable model is specified in Equation 2 as follows:

$$\beta_{1}wri_{it}+\beta_{2}X_{it}^{\prime }+\varepsilon_{it},$$ [Eqn 2]

for *i = 1, … N; t = 1, … T,* where *i* denotes country and *t* denotes time, and *ε* the error term. *X’* is a vector of mediating factor variables given by *X* = [*polity, kopen, rint, fdi, hcd*]. All the variables in the model are as defined in Table 4.

Several panel data estimation techniques address the above-mentioned characteristics of the dataset used in this study. The least square dummy variables (LSDV) with Driscoll and Kraay (1998) corrected standard errors is used in cases of both pooled regressions or individual effects, cross-sectional and time-specific effects, and moderate levels of temporal and CSD (Driscoll & Kraay 1998). It also applies when the error structure is heteroscedastic, and serially correlated within and between panels. The feasible generalised least squares (FGLS) technique developed by Parks (1967) and Kmenta (ed. 1986) is used to address individual effects, group-wise heteroscedasticity, serial correlation and CSD of the error term. The FGLS estimation technique is suitable whether the individual effects are fixed over time and cross-sections are normally distributed random variables. Feasible generalised least squares loses some efficiency in case of multiple sources of endogeneity (ed. Kmenta 1986). However, the Bruno (1995) correction that addresses Nickel (1981) bias and Generalised Method of Moments of Arellano and Bover (1995) with forward orthogonal deviations that address endogeneity from multiple sources did not yield meaningful results when applied in this study. This is because both estimation approaches that address endogeneity assume cross-sectional independence of the error term, which makes their results spurious because of the incidence of CSD in all estimations in this study (Baltagi 2005).

## Estimation results

The estimation results of Equation 1 are detailed in Table 6. It can be observed from Table 6 that in both models the coefficient of the disaster variable is negatively signed and statistically significant at 1% level.

**TABLE 6:** Growth model estimation. Dependent variable – GDP growth.

Variables	LSDV with Driscoll and Kraay (1998) corrected standard errors		Feasible generalised least squares (FGLS)	
	Coefficient	Standard error	Coefficient	Standard error
Natural disasters	−0.23***	0.05	−0.22***	0.04
Capital formation	0.13	0.26	−0.01	0.16
Human capital	0.10	0.11	0.16**	0.08
Urbanisation	0.38***	0.05	0.39***	0.05
Population growth	−0.38*	0.15	−0.39*	0.18
Constant	−4.56***	1.53	−4.07***	1.08
*R* ^2^	0.35	-	-	-
*F*-stat (prob.)	-	0.00	Prob. χ (5)	0.00

*Source*: Adapted from Driscoll, J.C. & Kraay, A.C., 1998, ‘Consistent covariance matrix estimation with spatially dependent panel data’, *Review of Economics and Statistics* 80(4), 549–560. https://doi.org/10.1162/003465398557825

LSDV, least square dummy variables.

* , 10% level of significance;

** , 5% level of significance;

*** , 1% level of significance.

This implies that disasters have a negative contemporaneous effect on economic growth in the countries studied.

As expected, human capital development and urbanisation have a direct relationship with economic growth in both models, denoted by their positive and statistically significant coefficients. This finding aligns with endogenous growth models that economies that are able to grow in the steady state are driven by research and development through human capital development. Additionally, urbanisation as a measure of industrialisation is expected to drive upward trends on economic growth (Solow 1956). Contrary to theoretical expectations, the results of both models reveal that gross fixed capital formation does not enhance economic growth in the countries in this panel. Population growth is negatively signed and statistically significant at 10% level. This is expected as an increase in the labour force without corresponding increases in productive resources leading to a decrease in marginal product of labour and ultimately growth.

In order to investigate the impact of time dimensions on the relationship between disasters and economic growth, we regress a one-period lag of the disaster variable on economic growth, *holding all other variables constant*. In other words, what is the relationship between a disaster a year ago and economic growth today. We also regress an interaction variable (*lwri* × *reconstruction*) on economic growth. The interaction variable consists of disasters a year ago, interacted variables that enhance reconstruction efforts. The interaction variable was constructed by principal component analysis in STATA. The aim was to establish the impact of a disaster a year ago, after reconstruction efforts, on economic growth today, a year after the incidence of the disaster. The results are detailed in Table 7.

**TABLE 7:** Bi-variate asynchronous estimation of economic growth and lag of natural disasters.

Variables	LSDV with Driscoll and Kraay (1998) corrected standard errors		Feasible generalised least squares (FGLS)	
	Coefficient	Standard error	Coefficient	Standard error
Lag_ natural disasters	0.72**	0.24	0.75***	0.12
Natural disasters* reconstruction	0.24**	0.07	0.10***	0.03
Constant	−0.23	0.17	0.13	0.12
*R* ^2^	0.21	-	-	-
*F*-stat (prob.)	-	0.00	Prob. χ (2)	0.00

*Source*: Adapted from Driscoll, J.C. & Kraay, A.C., 1998, ‘Consistent covariance matrix estimation with spatially dependent panel data’, *Review of Economics and Statistics* 80(4), 549–560. https://doi.org/10.1162/003465398557825

LSDV, least square dummy variables.

* , 10% level of significance;

** , 5% level of significance;

*** , 1% level of significance.

All two models report a positive and statistically significant relationship between disasters a year ago and economic growth, a year after the disaster occurs. This confirms that although the immediate impact might be negative, a year after the disaster the relationship turns positive. In addition, the coefficient of the interaction variable (*lwri* × *reconstruction*) is positive and statistically significant. This highlights the possibility that reconstruction and recovery measures if well-resourced could facilitate building back better that could ultimately lead to positive outcomes on economic growth a year after the disaster. This result is consistent with earlier research findings (Kliesen 1994; Skidmore & Toya 2002).

In order to test the hypothesis of Felbermayr and Groschl (2013), we estimate a third model regressing the disaster variable on institutional quality, financial openness, foreign direct investment, human capital development and real interest rate representing attractiveness to capital flows. The essence is to see whether these mitigating factors reduce a country’s level of risk to disasters as captured by the WRI. The results are shown in Table 8.

**TABLE 8:** Estimation results of mitigating factors. Dependent variable: natural disasters.

Variables	LSDV with Driscoll and Kraay (1998) corrected standard errors		Feasible generalised least squares (FGLS)	
	Coefficient	Standard error	Coefficient	Standard error
Lag_polity	−1.69**	0.60	−1.41***	0.33
Lag_financial openness	−0.63	0.35	−0.67**	0.27
Lag_real interest rate	0.06**	0.02	0.04***	0.11
Lag_fdi	0.15***	0.02	0.11***	0.02
Lag-human development	−0.05*	0.02	−0.06***	0.11
Constant	7.50***	0.35	7.63***	0.12
*R* ^2^	0.28	-	-	-
*F*-stat (prob.)	-	0.02	Prob. χ (5)	0.00

*Source*: Adapted from Driscoll, J.C. & Kraay, A.C., 1998, ‘Consistent covariance matrix estimation with spatially dependent panel data’, *Review of Economics and Statistics* 80(4), 549–560. https://doi.org/10.1162/003465398557825

Note: lag (.) represents the level of the mediating factor a year **before** the occurrence of the disaster.

LSDV, least square dummy variables.

* , 10% level of significance;

** , 5% level of significance;

*** , 1% level of significance.

A negative relationship between a mitigating factor and the disaster variable (depicted by a negative and statistically significant coefficient) would mean that the mitigating variable enhances the coping and adaptive capabilities of a country, thereby reducing its level of risk to disasters. The mitigating factors are lagged one period to investigate their impact at status quo should a disaster occur.

The results from the two models reveal that the coefficients of institutional quality, financial openness and human capital development are negative and statistically significant. This confirms the hypothesis of Felbermayr and Groschl (2013) that quality institutions, favourable financial conditions and adequate access to international markets enhance a country’s coping and adaptive capabilities to disasters, thereby reducing the country’s level of risk to disasters. The negative coefficient of human capital development variable further explains that research and development enhance a country’s coping and adaptation strategy, which reduces its level of risk to disasters. The interpretation here is that the more we know about disasters, the more we can adapt to surviving them and reducing their harmful impact. The coefficients of foreign direct investment and real interest rate are positive and statistically significant. A country that is attractive to capital flows attracts more foreign direct investment needed to recover and reconstruct after a disaster. In addition, countries recovering from disasters attract foreign direct investment in the reconstruction phase post-disaster. This has been the main concern of global policy dialogue on climate change, trying to address the cost of higher levels of industrialisation and growth to the environment and the price paid in terms of disasters experienced by many countries over the last three decades.

## Conclusion

This research study set out to explore the impact of disasters on economic growth in selected SADC countries. The common denomination amongst these six countries includes their natural proneness to disasters and the damages they generate. Annual data from the UNU-EHS and World Development indicators of the World Bank from 2005 to 2019 are used in this study. The data are estimated using dynamic panel data techniques that control for country- and time-specific characteristics, heteroscedasticity, serial correlation and CSD of the error term. Estimation approaches that fully control for endogeneity, such as the Nickel (1981) bias and GMM estimations, did not yield meaningful results. This is because of the incidence of CSD of the error term (Baltagi 2005).

The results of the estimations reveal that disasters do have a negative contemporaneous impact on economic growth in the countries studied. However, a year after the disaster the relationship between disasters and economic growth turns positive. This finds support from the literature that the impact of disasters on economic growth varies with time. The positive outcome on economic growth in a future period reflects the extent to which reconstruction and recovery measures could mitigate the initial negative damage caused by the disaster (Kliesen 1994; Skidmore & Toya 2002). Reconstruction and recovery measures that replace damaged capital stock with modern technology are known to positively impact economic growth. Reconstruction activity also generates increased sales tax receipts and additional employment (Kliesen 1994). However, the ability of a country to reconstruct post-disaster is further dependent on a number of mediating factors, which include the quality of a country’s institutions, degree of financial openness, human capital development and its ability to attract foreign direct investment, as confirmed by earlier studies (Felbermayr & Groschl 2013).

This positive net impact post-reconstruction finds empirical credence in the findings of this study. An interaction variable between the lag of disasters and factors that enhance building back better also showed a positive relationship with economic growth, a year after the disaster. Hence, consistent with Skidmore and Toya (2002), the net impact of disasters on economic growth is determined by the extent to which recovery and reconstruction post-disaster mitigates the initial negative impact of the disaster.

In terms of policy implications, countries need to ensure good institutional quality, which, in turn, attracts capital flows, human capital development through research and development, financial openness and foreign direct investment. These are crucial characteristics that facilitate the ability of countries to develop economic resilience towards disasters. The ongoing experience with a global disaster, such as coronavirus disease-2019, has shown how African countries having poor quality institutions and low levels of capital flows have struggled to manage the impact of the pandemic. Low levels of human capacity and research and development have hampered the ability of the continent to develop vaccines, ensure efficient contact tracing, testing for new infections and observing protocols that preserve lives. Massive fiscal constraints in mitigating the socio-economic impact of the pandemic have led to high levels of debt that average 70% of GDP on the continent. The ability of building back better in this pandemic and for any future disasters require that these mediating factors, degree of financial openness, institutional quality, attracting capital flows and human capital development, are at levels that can facilitate reconstruction and recovery from any disasters.
